# Using cascade CNN-LSTM-FCNs to identify AI-altered video based on eye state sequence

**DOI:** 10.1371/journal.pone.0278989

**Published:** 2022-12-15

**Authors:** Muhammad Salihin Saealal, Mohd Zamri Ibrahim, David. J. Mulvaney, Mohd Ibrahim Shapiai, Norasyikin Fadilah

**Affiliations:** 1 Faculty of Electrical and Electronics Engineering Technology, Universiti Malaysia Pahang, Pekan Campus, Pekan, Pahang, Malaysia; 2 Electrical Engineering Technology Department, Faculty of Electric and Electronic Engineering Technology, Universiti Teknikal Malaysia Melaka, Durian Tunggal, Melaka, Malaysia; 3 School of Electronic, Electrical and Systems Engineering, Loughborough University, Loughborough, United Kingdom; 4 Centre for Artificial Intelligence and Robotics, Malaysia-Japan International Institue of Technology, Universiti Teknologi Malaysia, Kuala Lumpur, Malaysia; Hanyang University, KOREA, REPUBLIC OF

## Abstract

Deep learning is notably successful in data analysis, computer vision, and human control. Nevertheless, this approach has inevitably allowed the development of DeepFake video sequences and images that could be altered so that the changes are not easily or explicitly detectable. Such alterations have been recently used to spread false news or disinformation. This study aims to identify *Deepfaked* videos and images and alert viewers to the possible falsity of the information. The current work presented a novel means of revealing fake face videos by cascading the convolution network with recurrent neural networks and fully connected network (FCN) models. The system detection approach utilizes the eye-blinking state in temporal video frames. Notwithstanding, it is deemed challenging to precisely depict (i) artificiality in fake videos and (ii) spatial information within the individual frame through this physiological signal. Spatial features were extracted using the VGG16 network and trained with the ImageNet dataset. The temporal features were then extracted in every 20 sequences through the LSTM network. On another note, the pre-processed eye-blinking state served as a probability to generate a novel BPD dataset. This newly-acquired dataset was fed to three models for training purposes with each entailing four, three, and six hidden layers, respectively. Every model constitutes a unique architecture and specific dropout value. Resultantly, the model optimally and accurately identified tampered videos within the dataset. The study model was assessed using the current BPD dataset based on one of the most complex datasets (FaceForensic++) with 90.8% accuracy. Such precision was successfully maintained in datasets that were not used in the training process. The training process was also accelerated by lowering the computation prerequisites.

## Introduction

Advancements in camera technology and the prevalence of social networks (Facebook, WhatsApp, and Instagram) and video-sharing sites (YouTube and Vimeo) have rendered digital media production, editing, and distribution easier and more popular. Processing requires each video frame to be altered individually, thus prolonging the fake video production process and time without sophisticated editing equipment and software. Consequently, realistic fake videos were rare as they are typically identified with the presence of explicit visual aberrations.

This situation has radically changed with the recent emergence of generative deep neural networks. The generative adversary networks (GANs) application [1, 2] has catalyzed the creation of software that limited manual intervention and produced videos from substantial photograph collections. The resulting fake media proved far more realistic when evaluated by a human viewer. As the first widely-available software implementing GANs, DeepFake was digitally published in early 2018. DeepFake, which employs GANs to replace an individual’s face in a video sequence with synthesized faces of another counterpart, witnessed a significant rise in the number of fake online videos involving a breach of privacy and identification and legal repercussions [1–7]. The necessity to detect such false videos has led forensic science community members to develop novel technology.

Conventional media forensic techniques have utilized signal level cues (double JPEG compression), physical level information, or semantic level consistencies (meta-data consistency). Nevertheless, such approaches were not sufficiently reliable or efficient in identifying more generic DeepFake videos [8–12]. Traditional contrast enhancement (CE) anti-forensic methods have depicted their practical forging ability in erasing the forensic fingerprints of enhanced images within histograms and the grey-level co-occurrence matrix (GLCM) with color filter array (CFA) interpolation using signal-level cues [13]. Regardless, the pixel value changes resulting from this approach are frequently exposed in the pixel domain. Latent GANs could be alternatively applied to mitigate this issue. The method outperformed deep-learning-based CE detection techniques in the pixel, histogram, and GLCM domains under anti-forensic attacks. Summarily, fake video detection with CFA is no longer deemed reliable [14, 15].

Adaptive PRNU denoising (APD) counter-forensic attacks on digital images did not previously affect image textural properties [16, 17]. Hence, Venkata et al. proposed an image-texture layer-based forensic solution for the source identification of APD counter-forensic images, which reported successful counter-forensic image source attribution with over 96% accuracy [18]. A calibration loss function could be applied to alleviate the variance gap in the high-frequency sub-bands between generated images and their calibrated versions to evade forensic detection [19, 20]. Following Jianyuan Wu et al., this method outperformed current advanced JPEG anti-forensic counterparts. In other words, fake image detection using double JPEG compression no longer proves suitable [21, 22].

Much emphasis has been placed on deep learning-based approaches and DeepFake countermeasures in addition to reviewing traditional media forensics methods. The attacker assumably modifies the metadata to render it useless as it would provide otherwise valuable information to verify image and video authenticity [14, 23]. Meanwhile, metadata are frequently omitted when media assets are uploaded to a social network. Thus, it is no longer deemed appropriate to rely on metadata consistency for authentication purposes [8, 24]. The current state of forensic approaches in terms of DeepFake video identification implies the urgent need for novel detection techniques. This research introduced a novel means of revealing DeepFake videos through the eye-blinking pattern of the synthesized face.

**Fig 1:**
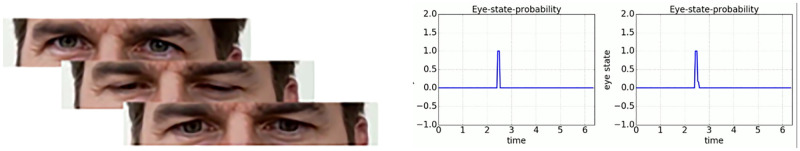
Explicit eye-blinking in real video sequences.

Blinking involves rapidly closing and opening one’s eyelid. The pre-motor brain stem controls these spontaneous blinks, which occur without conscious effort and serves as an essential biological function to (i) moisturize the cornea and conjunctiva with tears and (ii) remove irritants from the surface [25]. Generally, the interval between each blink is approximately two to 10 seconds for a healthy adult human with the actual rate varying based on the individual [26–28]. The duration of a typical blink cycle is between 0.1 and 0.4 seconds [11]. As such, spontaneous eye-blinking occurs within this specified frequency range and duration in videos of human. Contrarily, the core GAN model of DeepFake is trained with a large number of isolated human face pictures in many DeepFake videos. With an exposure time of 1/60 seconds, the probability of capturing a still image with the subject blinking is approximately 15% as illustrated in Fig 1. Based on the graphs, each action performed by the right and left eye proves useful to document any anomaly occurring throughout the target video. In reality, most of an individual’s online pictures would not depict closed eyes as such images were probably not selected for publication. The absence of eye-blinking is a useful characteristic to ascertain DeepFake media.

The current study proposed a method that potentially leverages the benefits of using a deep learning model to train blinking pattern features. A fully connected network (FCN) was utilized in this approach with processed datasets running into a trained long short-term memory (LSTM) network. Appropriate video pre-processing techniques were incorporated to reduce the FCN computation time.

The three study contributions are presented as follows:

1. The current work demonstrates how spatial and temporal information could be derived from the input eye sequence and the eye blinking probability could be computed by cascading convolutional neural networks (CNNs) with LSTM. The data, which are converted into meaningful knowledge by pre-processing the information into a labeled sequence, could be more easily employed by other researchers.
2. The eye-blinking pattern is utilized as a feature to detect anomalies in fabricated videos. The novel dataset facilitates the training process for classification purposes by minimizing the computation prerequisites and the memory required by GPU.
3. An FCN with three distinct models is applied to the classification stage. Each model is subjected to three separate tests entailing a range of epoch counts, patience values, and batch sizes. The optimal model and associated attributes are duly obtained.

## Related works

### DeepFake videos generators

Artificial images and videos are conventionally generated with detailed 3D computer graphics models. Goodfellow et al. [1] first proposed the use of GANs, which encompassed a network generator and discriminator. The generator employed a set of training images from which output candidate images were extracted for subsequent analysis by the discriminator. Both networks underwent training with the creator striving to generate images that could deceive the discriminating unit in its attempts to distinguish synthetic images from actual training ones.

Several articles that described picture or face synthesis methods through GANs have been published. Denton et al. [29] recommended a Laplacian pyramid GAN [30] for coarse-to-fine picture generation, whereas Radford et al. [31] who suggested deep convolutional GANs (DC-GAN) demonstrated the potential of such an approach for unsupervised learning. Meanwhile, Arjovsky et al. [32] utilized the gaps in Wasserstein distances to stabilize training. Isola et al. [5] employed conditional adverse networks to learn mappings from image to image and train the loss function while Shrivastava et al. [33] utilized an integration of adversarial loss and self-regularization loss to close a distance measure between artificial and real picture distributions. Liu et al. [2] proposed a coupled GAN-based unsupervised image-to-image conversion process to examine mutual image representations in several domains. Notably, this algorithm is the basis for that of DeepFake. The original face would be located while the landmarks for the whole face region would be defined to facilitate subsequent operations, such as accurate face-cropping to precisely warp the target face into the original. The face swap was subsequently applied to the original frame, which led to the creation of DeepFake.

### Detection of blinking eyes

The identification of eye blinks was previously examined in machine vision under fatigue detection applications [34, 35]. Pan et al. [25] developed an undirected conditional random field system to detect eye blinking by inferring eye closeness. The model simplifies the complex inference and optimizes the performance by omitting dependent eye state variables in a linear chain. Sukno et al. [36] employed active shape models with invariant optimal features (IOF-ASM) to delineate the eye contour and compute the vertical eye gap for eye condition assessment. A statistical analysis of the resulting shape sequence enabled the estimation of several blinking parameters. The outcome validation against manual annotations yielded a high level of accuracy in blink frequency estimation.

Yang et al. [27] incorporated a pair of parameterized parabolic curves to model the human eye shape and subsequently fitted a model to each frame in order to track eyelid movement. The face tracker, which was based on active shape models (ASMs) [37], employed a Kanade-Lucas-Tomasi tracker to continually track face landmarks. Determined by the face tracker, the eye region was refined by a deformable contour template for eyelid-fitting. A scalar quantity was proposed by Soukupova et al. [38], in which a rectangular bounding box was placed around the eye with an aspect ratio paralleling the degree of eye openness. The elicited pattern relied on the speed of eye-closing and opening, degree of eye-squeezing, and blink duration. In this vein, an ‘eye aspect ratio support vector machine’ was developed to identify the final eye condition with these features as inputs.

Kim et al. [6], who studied CNN-based classifiers to assess if one’s eyes are open or closed, employed a ResNet-50 revised model [39] with a specified number of nodes in its fully connected layer. Li et al. subsequently extended the CNN-based classifier to consider the temporal relationship between consecutive frames as eye blinking occurs over several frames. Long-term recurrent convolutional networks proved suitable to analyze changes over several frames while mitigating the significance of changes introduced between consecutive images [40]. The authors then extended the method with LSTM-RNN to better control when and how to forget previous and update the currently hidden states.

## Methodology

**Fig 2:**
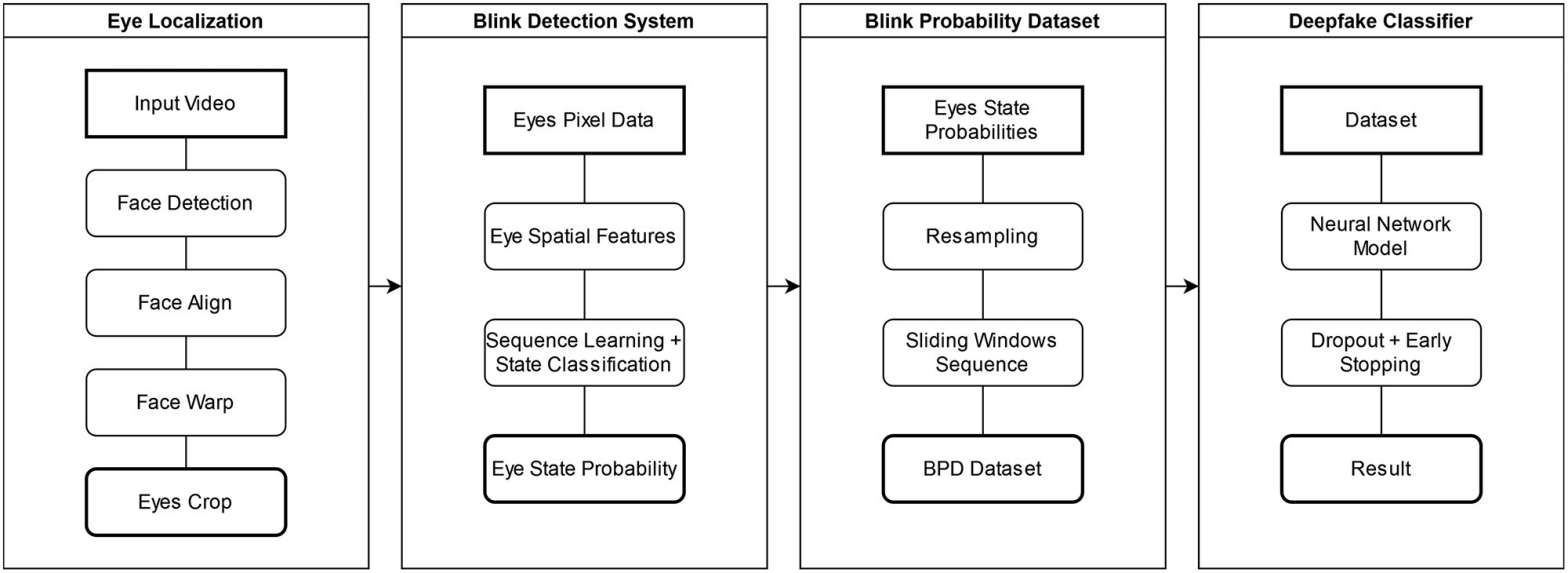
DeepFake detection approach based on eye state data.

Fig 2 below depicts the DeepFake detection approach, whose process can be divided into the following stages:

- Stage 1: Eye Localization. The video is extracted into frames during the pre-processing period with each face in each frame being characterized. The recognized faces are subsequently aligned and warped to ensure the consistency of their eye orientation and direction. The eyes are then cropped and saved as pixel values.
- Stage 2: Blink Detection System. The characteristics-extracting method allows the status of both eyes to be recognized. Every frame series is fed into the cascade model, which entails convolution, LSTM, and an FCN. Essentially, the output represents the probability of each eye condition.
- Stage 3: Blink Probability Dataset (BPD): The probability data collected in Stage 2 are re-sampled and processed within 4.5-second sliding windows to develop a novel BPD dataset.
- Stage 4: DeepFake Classifier. This classifier functions to train real and fake videos with data from the BPD dataset on eye state probability. The FCN architecture model is selected for training together with its dropout, early-stopping, batch size, and network configuration.

The visual studio code-Python integration served to run the algorithm. The OpenCV library was incorporated for frame extraction from the videos and image manipulation. Meanwhile, dlib library was applied to recognize the face mark, which allows bending, warping, and cropping of the eye. Tensorflow functioned as a framework to run the algorithm during the eye-blink state extraction based on a pre-trained model. This extraction was utilized to develop the blink pattern from the BPD dataset. A further model was structured following this new dataset to predict DeepFake videos with PyTorch and PyTorchLightning. Both Tensorboard and matplotlib were employed for outcome virtualization.

### Eye localization

**Fig 3:**
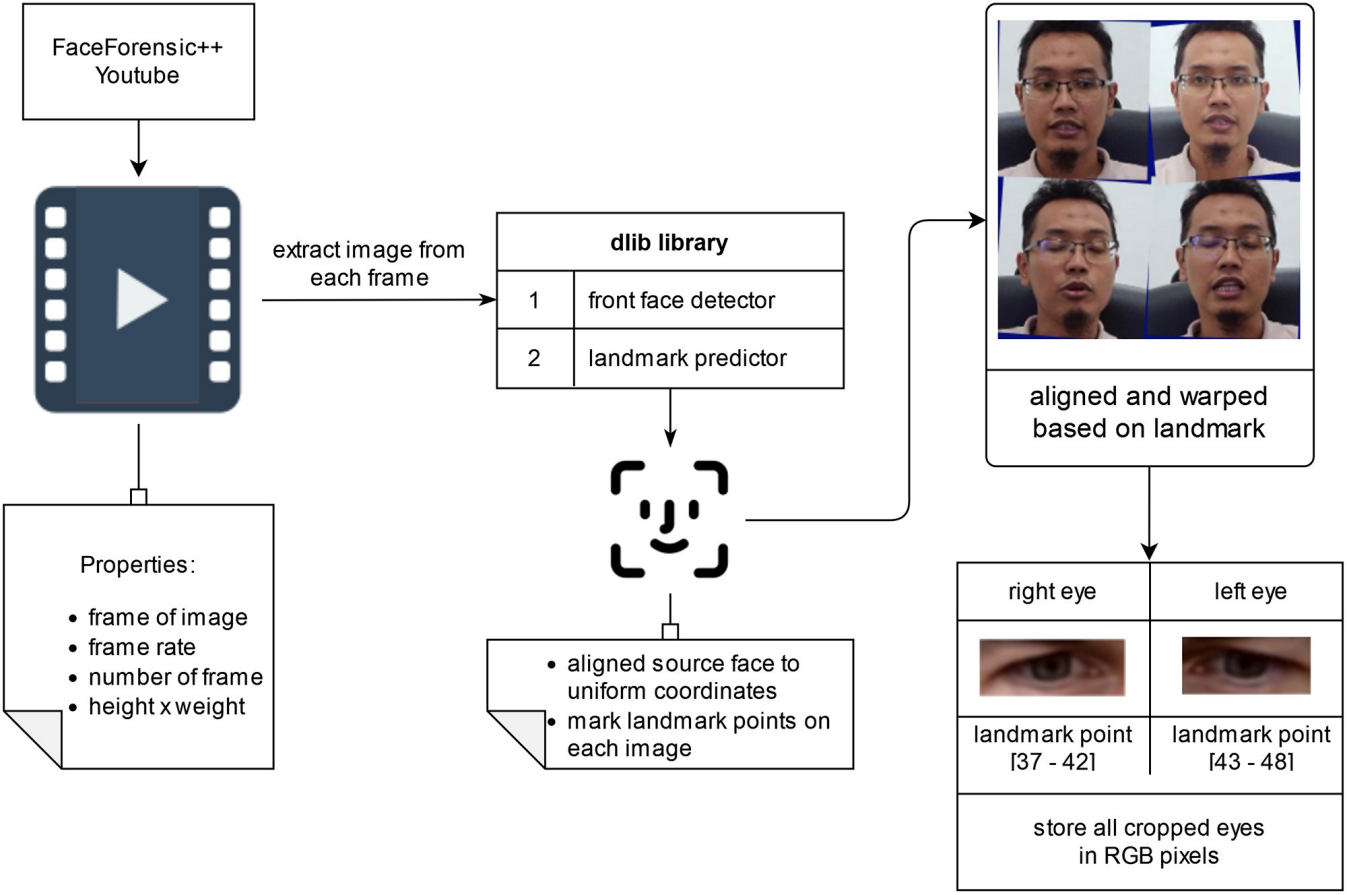
Obtaining the eyes from the input videos.

First, the datasets provided by Zhou T, Wang W, Liang Z, et al., the FaceForensics++ dataset [41] and YouTube videos were extracted into individual frames. The face is detected through the dlib library while the landmark of the face is duly determined. The function of the library and FL detectors depend on the approach represented by [42], which outputs an array of 68 points in the (x, y) coordinate format. Essentially, the faces are aligned and warped before the eyes are cropped into a single input to ensure that the line joining the eye centers is horizontal and scaled to a uniform size. The eye positions are determined by points 37 to 48. Furthermore, the rectangular region is established by omitting the bounding boxes of each eye landmark points, thus scaling each bounding box by a factor of 1.25 in the horizontal direction and a factor of 1.75 in the vertical direction and ensuring the inclusion of the eye region in the cropped zone. Fig 3 illustrates the relevant processes. The depicted individuals provided informed consent in written form based on the PLOS consent form to publish their image alongside the manuscript.

### Blink detection system

**Fig 4:**
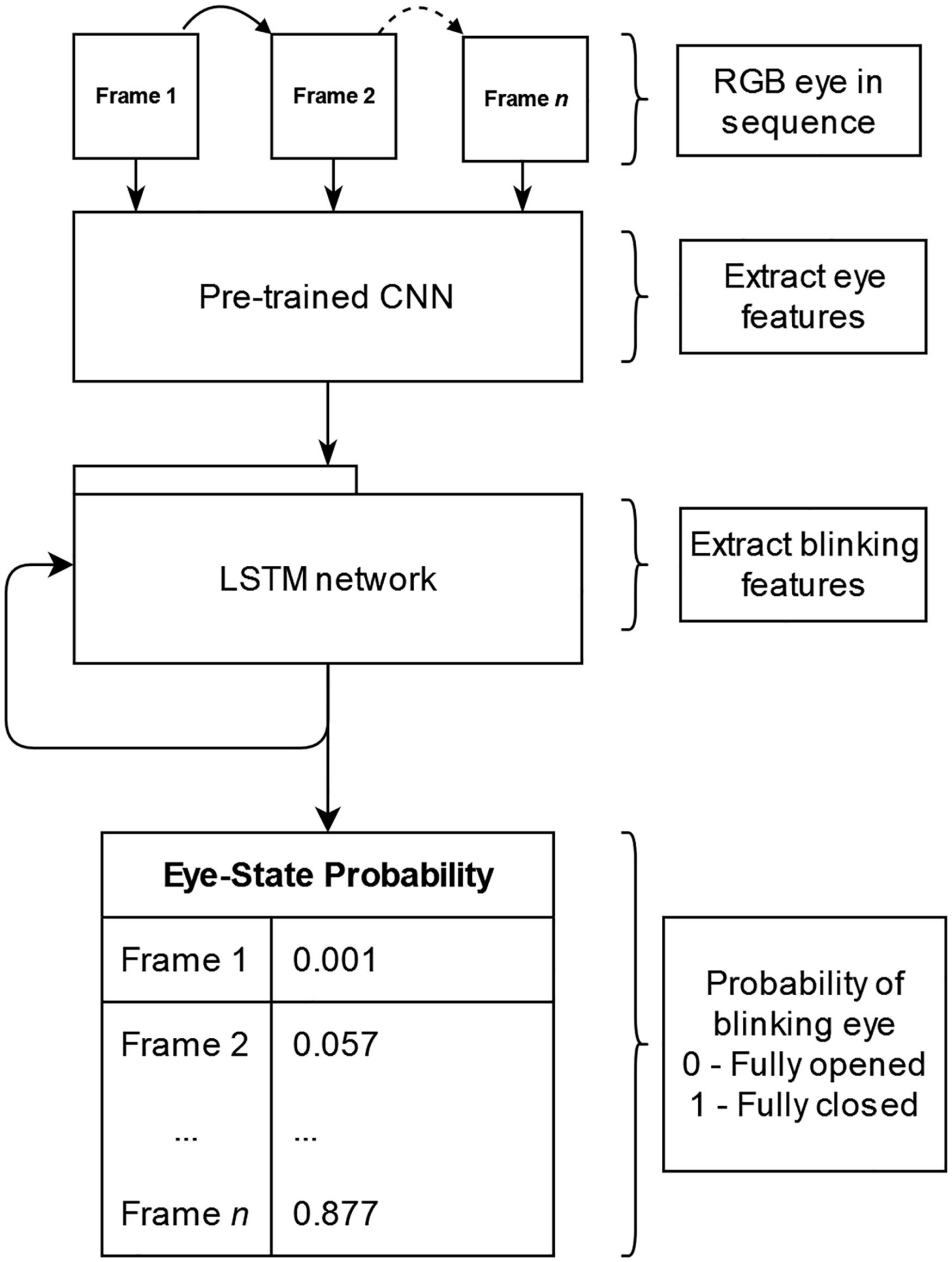
Blink detection system.

Fig 4 illustrates the blink detection system performance procedures. The sequences encompassing the cropped eye regions, which were derived from the eye localization in Stage 1, were saved as RGB images. These data sequences were fed into a pre-trained CNN to extract the spatial information for each eye. Additionally, the extracted features were fed into the LSTM network for further feature extraction.

**Fig 5:**
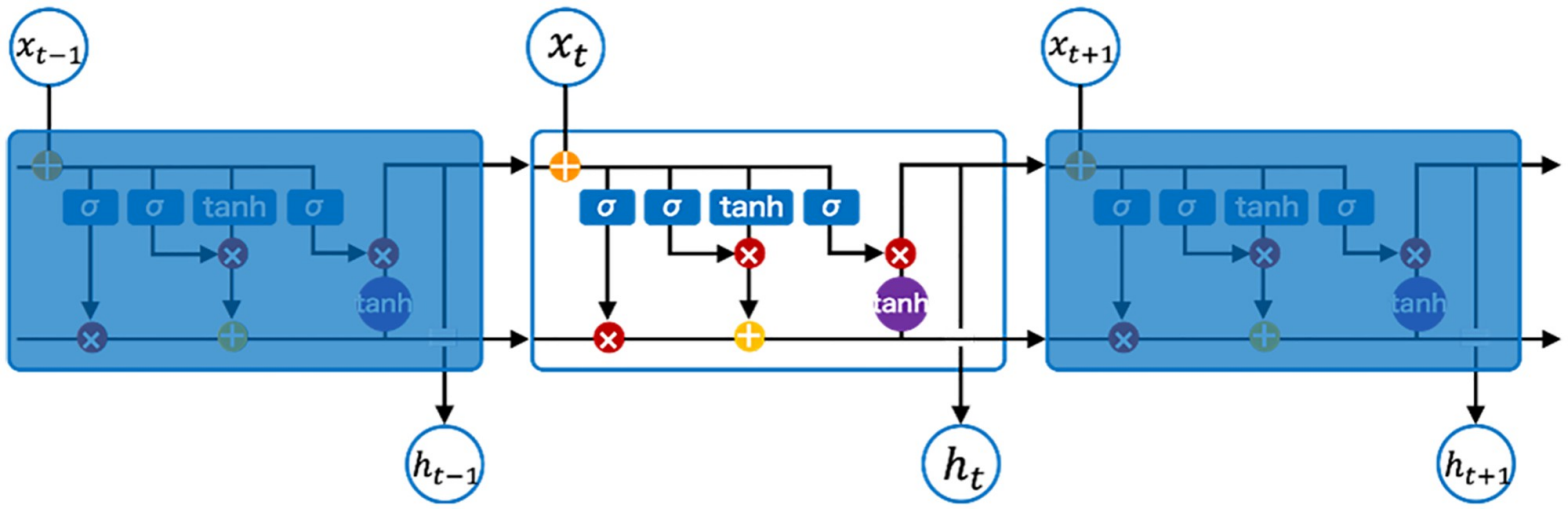
The LSTM.

The LSTM nodes illustrated in Fig 5 denote memory units that regulate when and how (i) previous hidden states are forgotten and (ii) hidden states are updated [4]. The first sigmoid function from the left of the block diagram, which is known as the forget gate, *f_t_*, pushes the input *x_t_* into the [0, 1] range.

$$\begin{array}{c} f_{t}=\sigma \left(w_{f}\cdot \left[h_{t-1},x_{t}\right]+b_{f}\right) \end{array}$$ (1)

where *σ* denotes sigmoid function, *w_x_* implies the weight for respective gate(x) neurons, *h_t_* reflects the LSTM block output, and *b_x_* indicates the biases for the respective gates(x).

The vector output of the forget gate was simply formed from a dot product of the input weights and previous cell states. The result would ascertain whether to amplify or attenuate the original value. As a cross product with the hyperbolic tangent function of previous cell states, the second sigmoid function constrains the input to the [−1, 1] range.

$$\begin{array}{cc} & i_{t}=\sigma \left(w_{i}\cdot \left[h_{t-1},x_{t}\right]+b_{i}\right) \end{array}$$ (2)

$$\begin{array}{cc} & {\tilde{C}}_{t}=tanh\left(w_{c}\cdot \left[h_{t-1},x_{t}\right]+b_{c}\right) \end{array}$$ (3)

$$\begin{array}{cc} & C_{t}=f_{t}*C_{t-1}+i_{t}*{\tilde{C}}_{t} \end{array}$$ (4)

where ${\tilde{C}}_{t}$ implies the candidate for cell state at timestamp(t), *C_t_* denotes the cell state at timestamp(t), and other mirror Eq 1.

The sigmoid output (amplifier or attenuator) was subsequently utilized to scale the encoded data based on its appearance pre-application to the cell state. Plausibly, the inclusion of such features to render the present state essential to recall implies their reference as input gates in (Eq 1), which would later be integrated with the forget gate from (Eq 3) to form the new cell state (*C_t_*) as expressed in (Eq 4). A hyperbolic tangent was applied to the new cell state in compressing the values into the [-1, 1] range as presented in (Eq 5). Lastly, the cross-product with the input sigmoid function was expressed in (Eq 6).

$$\begin{array}{cc} & o_{t}=\sigma \left(w_{o}\cdot \left[h_{t-1},x_{t}\right]+b_{o}\right) \end{array}$$ (5)

$$\begin{array}{cc} & h_{t}=o_{t}*tanh\left(C_{t}\right) \end{array}$$ (6)

where *o_t_* represents the output gate and *h_t_* depicts the LSTM block output at timestamp(t).

This outcome formed the current recurrent network output, which also became the hidden state for the subsequent network. At this point, the LSTM model output provided the temporal features derived from the sequence of cropped eyes. Regarding the final prediction state, each LSTM neuron output was sent to a neural network constituting a fully connected layer, which incorporated the LSTM output and generated the probabilities of both open and closed eye states.

### Blink Probability Dataset (BPD)

The eye state probability values gathered in the Blink Detection System section were stored in a temporal sequence that paralleled the input source frame rate. Each video sequence encompassed a modified frame rate and duration with the probability sequence for each video also undergoing changes. As such, it proves necessary to standardize the frame rate for the blinking pattern to be used as a legitimate feature in system training and the incoming data to have a specific length of sequence before proceeding. An input sequence that is too long would require trimming within a particular sliding window formation. The frame rate for each probability sequence was converted to 50 frames per second. Any data in between were resampled using the value from the prior data. As a result, the sliding windows are set at 4.5 seconds, resulting in 225 eye-state probabilities in each sequence, so ensuring that there is the opportunity for the eyes to blink at least once within the trimmed sequence.

**Fig 6:**
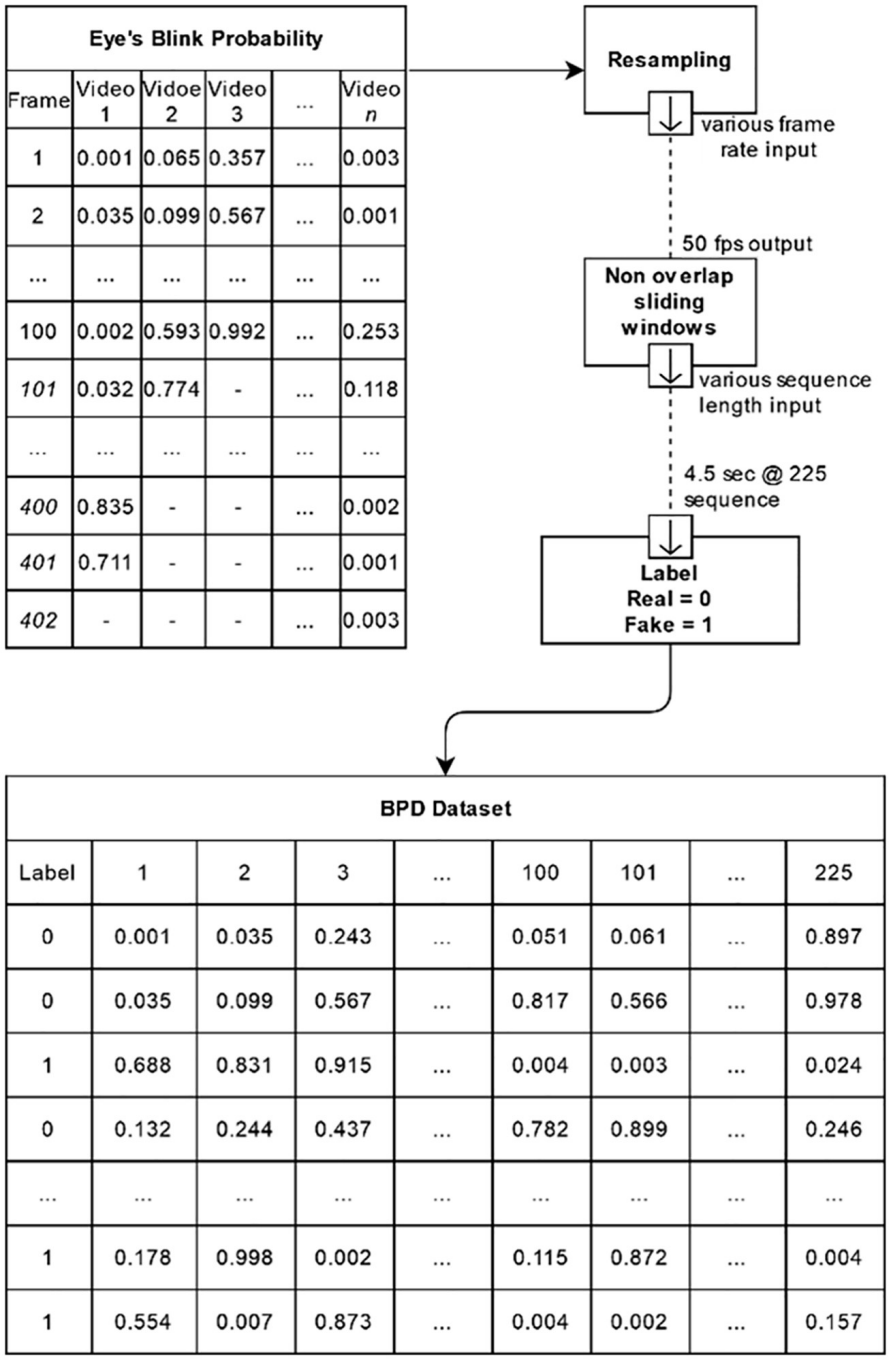
Processing the raw data into a complete BPD.

The final (BPD) dataset had the input marked with a 0 for an authentic video and 1 for a tampered one (see Fig 6). Each input constituted 225 features ranging from the second to the 226^th^ column. These properties stored the probability of blinking with values ranging between 0 (eye fully open) and 1 (eye fully closed). The 225 probability values reflected the eye condition for 4.5 seconds, during which at least one eye blink would normally occur. As stated in the final stage, the whole dataset was fed into the study models for preparation, validation, and testing purposes.

### DeepFake classifier

An FCN is typically defined to provide appropriate discrimination of features. Specifically, the FCN output denotes a predicted classification label. All inputs pass through the fully connected layer with a separate weight applied for each connected neuron. Selecting the most optimal combination of layers would offer an optimum network with minimal calculation cost.

The operations performed by the FCN are presented below:

$$\begin{array}{cc} & n_{0}^{out}=I \end{array}$$ (7)

$$\begin{array}{cc} & n_{0}^{in}=n_{0}^{out}*W_{i}+B_{i} \end{array}$$ (8)

$$\begin{array}{cc} & n_{i}^{out}=F_{i}\left(n_{i}^{in}\right) \end{array}$$ (9)

where *F_i_* denotes an activation function for layer *i*. With this formula, a forward pass could be executed and the network output produced for each network layer.

Backpropagation was then applied to update all the weights and biases. The weight and bias update process in this study involved Diederik and Jimmy et al.’s adaptive moment estimation [7], whereas stochastic gradient descent served to update the neural network parameters. Meanwhile, adaptive gradients (AdaGrad) offered a direct means of gradually varying the learning rate to accommodate dataset changes, as small- or large-scale shifts are possible based on how the learning rate is selected. The equation is expressed as follows:

$$\begin{array}{c} \theta_{t+1,i}=\theta_{t,1}-\frac{\eta }{\sqrt{\epsilon +\sum_{\tau =1}^{t}{\left(\nabla J\left(\theta_{\tau ,i}\right)\right)}^{2}}}\nabla J\left(\theta_{t,i}\right) \end{array}$$ (10)

where *θ* implies a parameter consisting of the weight, biases and activation,*η* reflects the learning 258 rate, ∇ denotes the gradient, and *J* is the objective function with its features and labels.

To initialize the calculation of the error gradients, it is necessary to provide an error calculation for determining the losses. This work uses the cross-entropy loss as it is widely adopted by researchers in this field. The cross-entropy loss *L* is given by

$$\begin{array}{c} L=-\sum_{j}^{C}y_{j}\text{ln}{\hat{y}}_{j} \end{array}$$ (11)

where *C* represents the number of classes, *y* denote the labels, and ŷ imply the predicted labels.

The loss, which dictated the gradient of the backpropagation steps, was updated based on the selected learning rate. A batch of inputs were fed into the network during each epoch with the weights and biases updated at the end of the epoch. The final accuracy and loss value were computed as FCN performance measures.

Based on the newly-established BPDs, each input constitutes 225 features reflecting the eye blinking state every 0.02 seconds, thus providing a total duration of 4.5 seconds for the eye sequence. It is designed so that at least one eye blinking condition would be present in each sequence. The sequences were fed to FCN networks with a range of different layer designs, each with a 0.1 dropout. The rectified linear unit (ReLU) selected as the activation function was applied at the output of each layer. The probability used in the prediction of the video ingenuity status was provided by the output layer values.

The process for distinguishing between genuine and tampered videos in this research was derived by feeding the BPD into three different FCN architectures. In line with Table 1, each model has its own number of hidden layers and individual number of nodes in each layer.

**Table 1:** Layers of FCN models used.

Layer	input	*h*_1_	*h*_2_	*h*_3_	*h*_4_	*h*_5_	*h*_6_	output
*Model*1	225	512	1024	256	64			2
*Model*2	225	512	1024	128				2
*Model*3	225	512	1024	2048	1024	512	256	2

Other than the models used for testing purposes, each model was executed with a distinct set of settings and parameters. The number of epochs in Experiment 1 was set to 100, 300, and 500. Experiment 2 incorporated an early stopping function into each model with patience values of 20, 40, 60, 80, and 100, whereas experiment 3 demonstrated the result of adjusting the batch value from 5, 10, 15, 20, and 25 to achieve the highest precision for the epoch setting derived from experiments 1 and 2. All the experiments assessed the model accuracy with the outcomes and the analysis elaborated in the following section.

## Results and discussion

The current study datasets were established through the FaceForensics++ dataset and converted into probability values for the eye-blinking state, which were subsequently trimmed to fit the required 225 values of the input data sequence. This new data collection trained the model and validated the classification of real and fake videos. This experimental study encountered several limitations. For example, videos with more than one individual were excluded as the method only analyzed one face at a time. The selected face was then pre-processed with only the left and right eye areas included in the training model. The dataset source was elicited from the FaceForensics++ dataset. Furthermore, five different tools involving DeepFake, DeepfakeDetection, Face2Face, FaceSwap, and Neural Textures were employed to generate the Deepfake media although the media source was restricted to a single dataset. In this vein, the high levels of diversity and complexity were regarded In the Wild dataset.

### Dataset

A total of 451 videos consisting of 200 real videos and 251 tampered ones were generated. Each video represented one individual with at least one blink occurring to form the BPDs. The datasets were prepared with Yuezun Li et al.’s [11] annotation tools where each video provided two CSV files with timestamps determined based on video length and frame per second (fps). These files were subsequently processed to form part of the final BPD dataset.

### Eye blinking extraction result using CNN-LSTM pre-trained model

**Fig 7:**
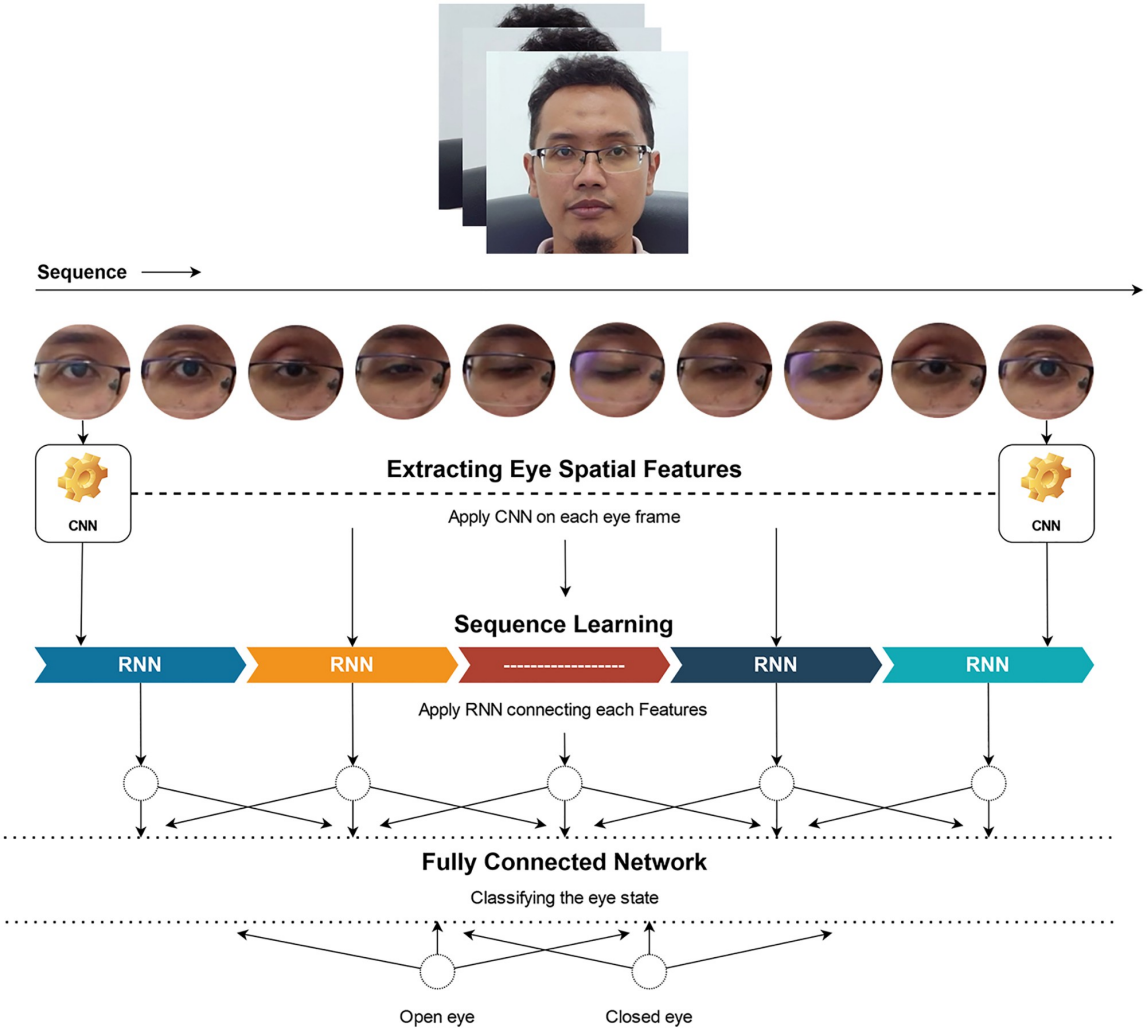
Pre-trained CNN-LSTM model for eye state prediction.

Each video was analyzed before being fed to the proposed model. The pre-trained CNN-LSTM model omitted the eye blinking state for each frame. Specifically, the model predicted a probability value for every (open/closed) state for each eye and frame in each clip. Fig 7 illustrates the model operation. The depicted individuals in Figs 7 and 10 had provided written informed consent in compliance with the PLOS consent form to publish their image alongside the manuscript.

**Fig 8:**
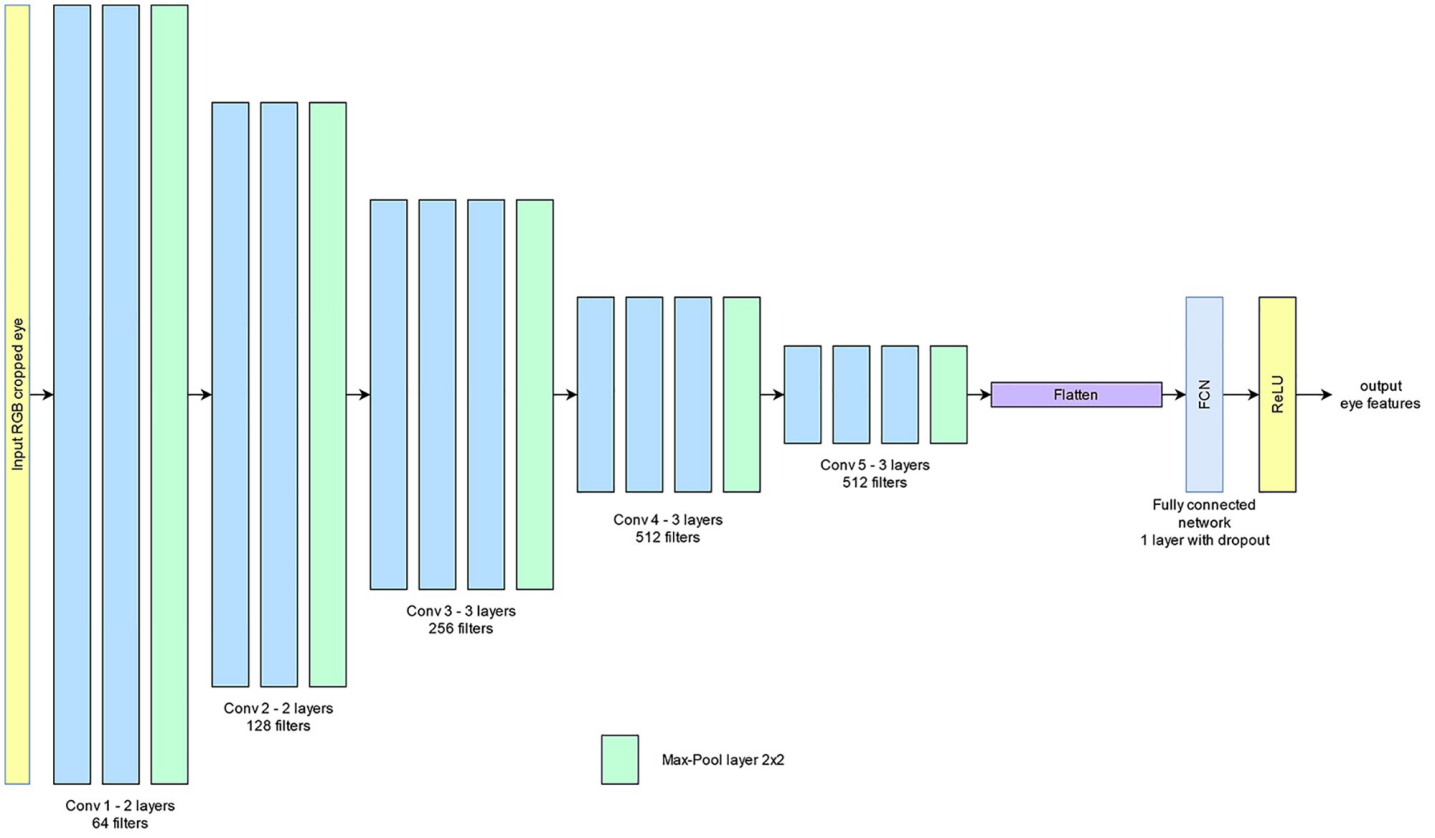
The VGGNet-16 model for extracting eyes spatial features.

The CNN used in this work is based on VGGNet-16 model (see Fig 8) and comprised 16 convolutional layers. Pre-training was performed on the model with ImageNet datasets. This model was selected given its outstanding precision in eliminating features from still images and ability to distinguish between apparent changes in the eye size when opening and closing.

**Fig 9:**
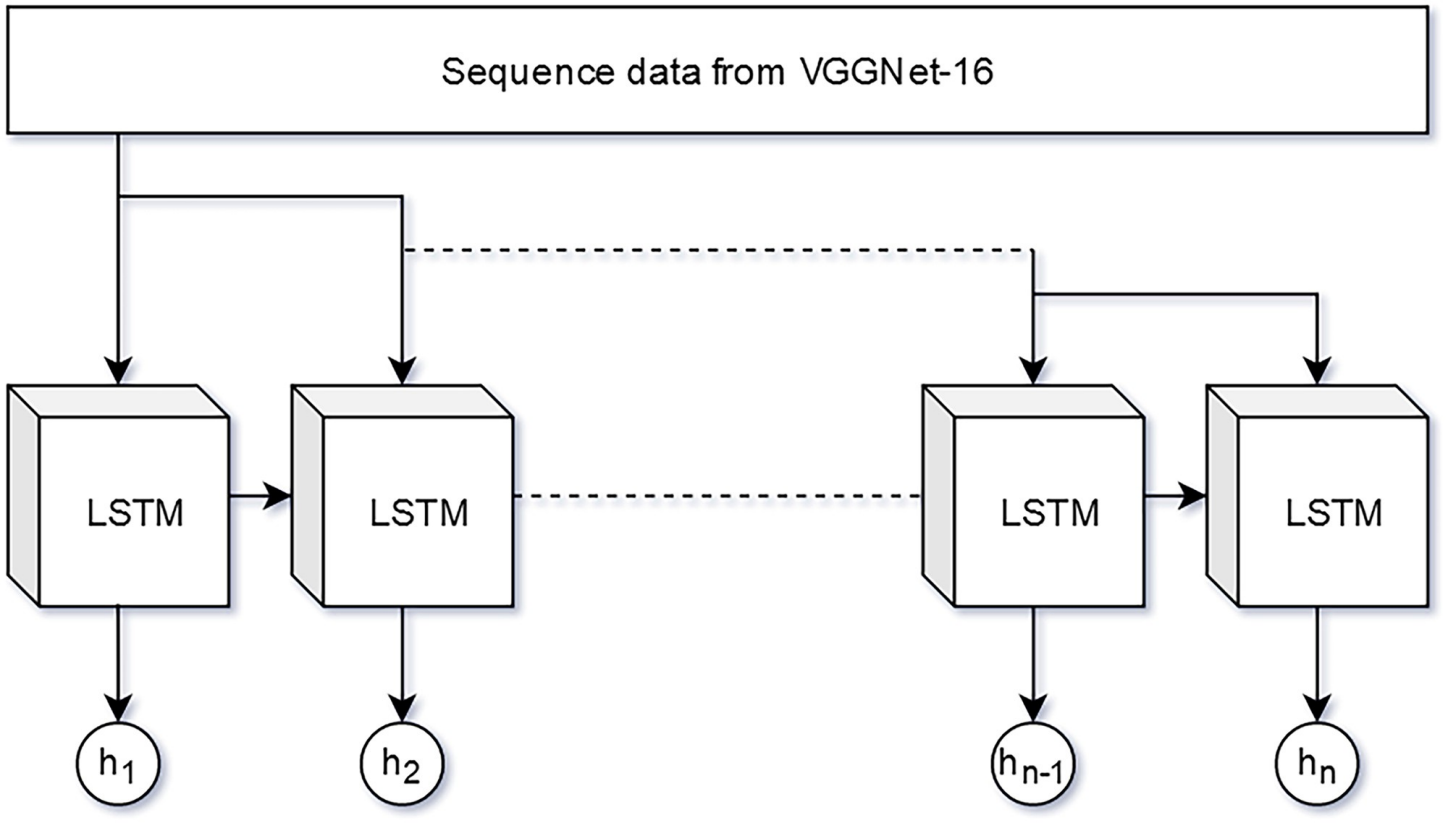
The LSTM network for extracting temporal features of eye-blinking state.

A technique with sole reliance on CNN to train the pixel pattern of the eyes and the system is generally incapable of ascertaining whether the eyes were in the closing or opening state. Predictions on changing eye state could be improved through an RNN that incorporates temporal features. The CNN output was reshaped into 20 sequences and fed to a RNN with the LSTM variant. Notably, LSTM could extract the relationship between the temporal features of the sequences for each set of the 20 spatial features elicited from CNN. The performance measures derived for the many-to-many LSTM network was routed through FCN for classification. Fig 9 illustrates the complete architecture.

A technique that relies solely on CNN to train the pixel pattern of the eyes and the system is often unable to distinguish whether the eyes were in a state of closing or opening. The prediction of changing eye state can be improved by using a RNN that incorporates, temporal features.

**Fig 10:**
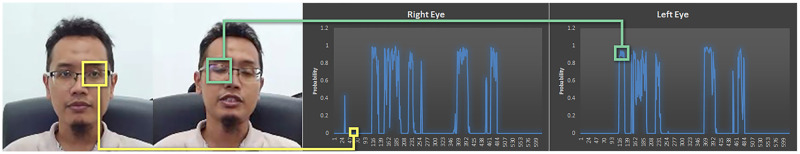
Result of CNN-LSTM network for every frame in the video input.

The video signal, which was fed into the network, yielded a probability value for the eye state ranging between 0 (eye fully open) and 1 (eye fully closed). In line with Fig 10, the CNN-LSTM network precisely predicted the eye probability that represented the eye-blinking status on both sides.

**Fig 11:**
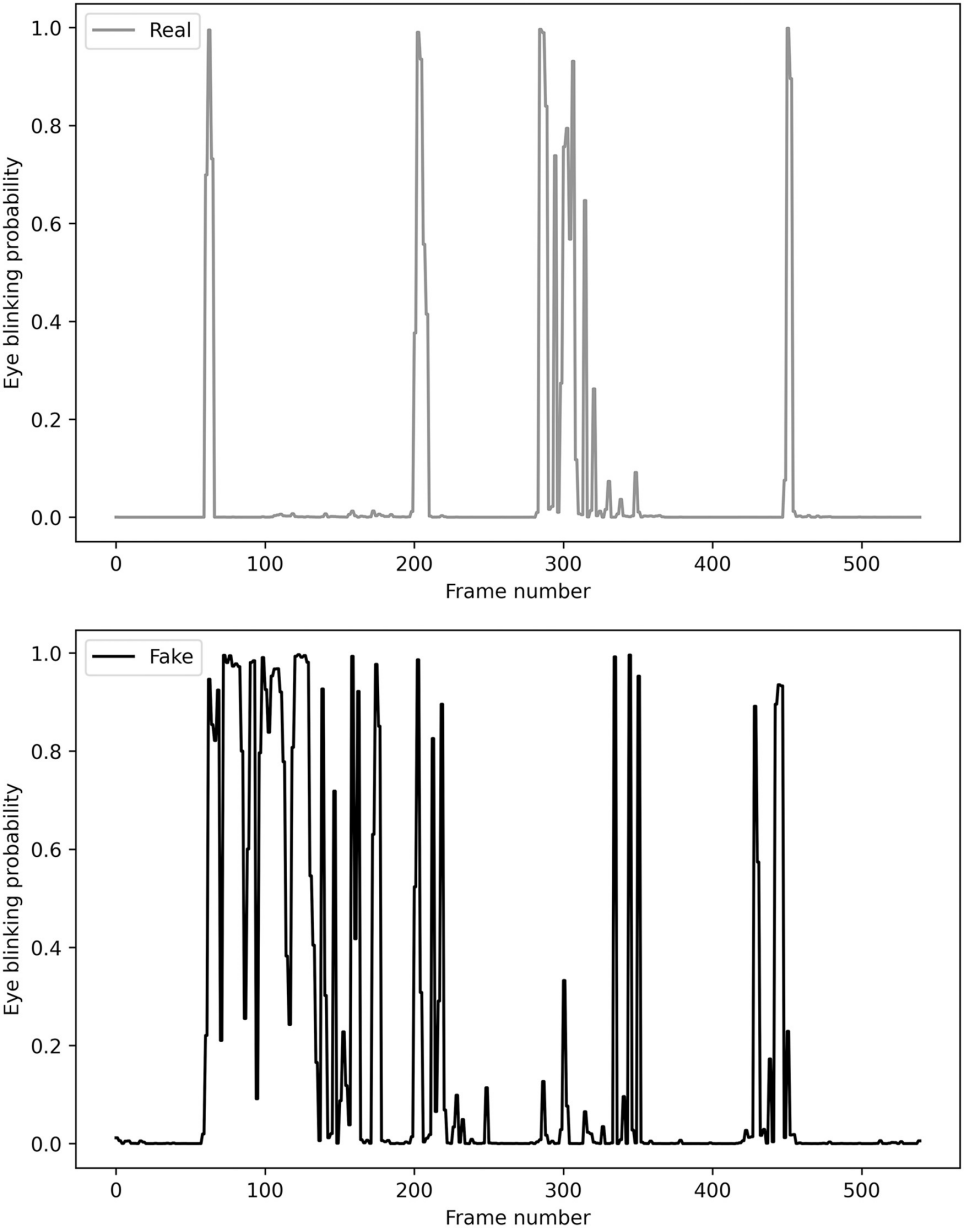
Sample of blink sequence pattern for real and fake video.

Fig 11 depicts an individual’s blinking sequence from the original video versus the DeepFake-generated counterpart. Observably, the generated eye sequence between opening and closing proved intermittent: an unnatural blinking pattern compared to the original sequence. The anomaly in the fake video was amplified by the temporal features of the blinking sequence and fed into the train model.

### Classification

The BPD dataset elicited in the Dataset section was fed into the model for training purposes. Particularly, the dataset was divided into (i) training-evaluating and (ii) testing sets. The training-evaluation set encompasses 3484 data values for both real and fake videos with 70% of the set utilized for training, 15% for validation and 15% for testing. The *train*, *validate*, and *test* Dataloaders were then distributed using a random split. Dropout with a value of 0.1 was used for each neural network layer to avoid overfitting cases. The learning rate of 0.0004 was selected with ADAM incorporated as the optimizer. Meanwhile, the random seed was set to 42 in each epoch in standardizing the initial weight and demonstrating a valid comparison.

The input data length was fixed at 225 data points during training. Each hidden layer passes was fully connected, albeit with a different number of nodes and a specific rectified linear activation function. The FCN generated a SOFTMAX output that implied the media authenticity or fakeness. Several training cycles were attempted with various batch sizes, epoch counts, and early stopping mechanisms. The training model was evaluated for each set of experiments by considering the *test_acc* and *test_loss* derived from the trained model. Notably, this model employed the same dropout value and activation mechanism, albeit with differing layers between the study models (see Table 1).

#### Experiment 1: Epoch

The first experiment was run with a batch size of 15 and no early stopping. Nevertheless, each model was trained for 100, 200, 300, 400, and 500 epochs. Table 2 presents the elicited outcomes.

**Table 2:** Test accuracy for a range of epoch values in three FCN models.

Number of Epoch	100	200	300	400	500
*Model*1	82.18%	84.29%	83.52%	**88.31**%	86.97%
*Model*2	82.76%	84.10%	84.11%	85.82%	82.57%
*Model*3	80.65%	84.29%	84.10%	86.20%	83.14%

Based on the test dataset accuracy, all the models could deliver over 80% of accuracy with Model 1 demonstrating the highest at 88.31% when predicting real or fake films through 400 epochs. Both Models 2 and 3 also performed optimally with 400 epochs. Although Model 3 depicted the most hidden layers with prolonged training time, it failed to provide a significantly improved performance.

#### Experiment 2: Early stopping

The second experiment was conducted by fixing the batch size at 15. Regardless, the early stopping incorporated into each model stopped at a specific number of epochs upon meeting the final condition. The *patience* values used were set at 20, 40, 60, 80, and 100. Table 3 presents the elicited outcomes.

**Table 3:** Test accuracy for early stopping with patience setting of 20, 40, 60, 80, and 100 on three FCNs models.

Patience	20	40	60	80	100
*Model*1	81.61%	83.72%	84.29%	85.05%	**89.66**%
*Model*2	81.42%	79.89%	74.33%	81.99%	81.23%
*Model*3	79.69%	83.33%	83.14%	83.33%	83.33%

Although the early stopping function rapidly completed the training, the degree of rapidity depended on the validation accuracy value requirement. This approach could generate a better model with minimal training time. For example, Model 1 demonstrated an outstanding result of 89.66% in test accuracy when performed with a patience value of 100, whereas Models 2 and 3 failed to attain better accuracy in any patience setting. It is deemed possible to cease training early when a large neural network could generalize in a manner comparable to a smaller counterpart. This ability could efficiently minimize calculation time and generate optimal performance.

#### Experiment 3: Batch size

The final experiment fixed the epoch number that provided the most optimal outcomes in Experiments 1 and 2. The employed batch sizes were 5, 10, 15, 20, and 25 (see Table 4).

**Table 4:** Test accuracy for a batch size of 5, 10, 15, 20, and 25 at best epoch number for each FCNs models.

	Batch Size Epoch	5	10	15	20	25
*Model*1	105	90.04%	90.61%	89.66%	**90.80**%	89.85%
*Model*2	400	86.59%	85.25%	85.82%	84.67%	84.67%
*Model*3	400	85.44%	86.78%	86.20%	86.78%	86.59%

With a batch size of 20 and 105 epochs, Model 1 offered the most optimal accuracy value of 90.80%. All the models denoted little variation in terms of test accuracy. As the number of connected layers was reduced, the model could rapidly achieve convergence to better minima. Larger batch sizes expanded the training to include additional compute nodes, which would save energy in reduced computation efforts.

**Fig 12:**
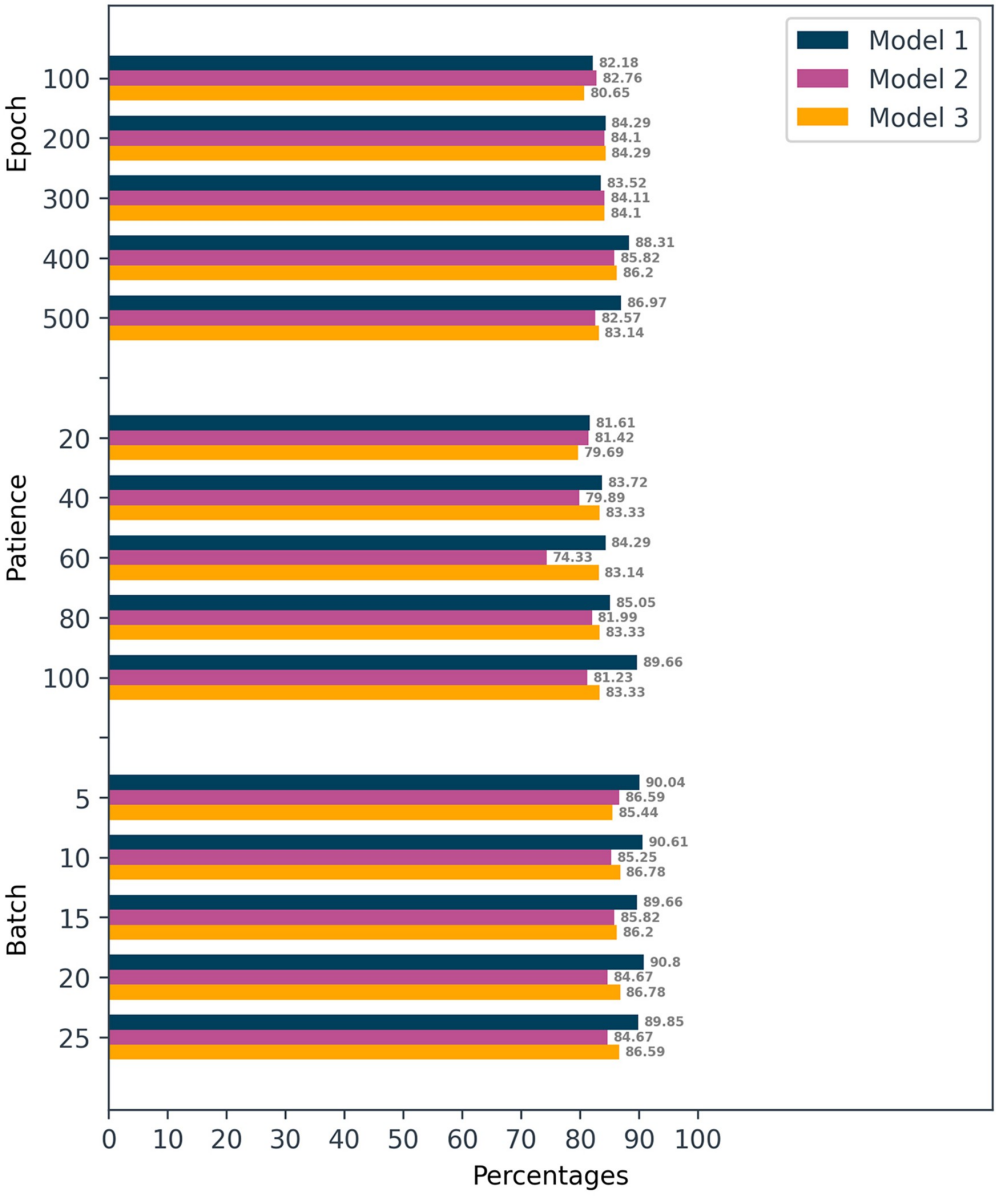
Comparative performance of the three models.

Fig 12 presents a summary of the experimental outcomes. Despite having fewer hidden layers than other models, Model 1 reflected the most optimal performance in detecting fake video sequences. Establishing early stopping as an extra callback enables specific models to end the learning process earlier, alleviate training costs, and minimize time consumption. Notwithstanding, the end model in this study relied on an FCN that rendered early stopping ineffective as observed in Experiment 2. Selecting a specific batch size could significantly improve accuracy while saving time during the training session. Following the results derived from all three experiments, Model 1 with a batch size of 15 offered the highest accuracy without depending on an early stopping mechanism.

Table 5 presents several approaches to the same problem. Using only spatial information, Guo et al.’s incorporation of AMTEN and CNN provided 87.05% accuracy post-testing on their own datasets [43]. This accuracy significantly improved when RGB information was utilized as input along with the noise features extracted by a spatial rich model, hence resulting in 90.36% accuracy assessed on a still image in the FaceForensics++ dataset [44]. Regardless, insufficient temporal information deterred the model from performing optimally on sequence data. Integrating temporal information as additional features enables one to examine the inconsistency between frames as the change occurs at the single frame level. Although the performance of [45] and [47] on the FaceForensics++ dataset proved slightly lower than the spatial-based technique, merely utilizing the basic model to extract both spatial and temporal features could still provide a substantial outcome with accuracy up to 85.80% [46].

**Table 5:** Performance comparison with other detection technique.

Dataset	Architecture	Classifier	Strategy	Accuracy
Face RGB and Own Dataset	AMTEN with CNN [43]	FCN	Spatial	87.05%
Face RGB FF++	2 stream XceptionNet (pixel & filtered) [44]	FCN	Spatial	90.36%
Face RGB, FF++	Optical flow feed into ResNet50 [45]	FCN	Temporal	80.56%
Face RGB, FF++, Deepfake-TIMIT dataset	Use spatial angle and temporal rotation as classifier input [46]	SVM	Spatial Temporal	85.80%
Face RGB seq, CelebDF, FF++ dataset	2 stream: MesoNet + ResNet [47]	FCN	Spatial Temporal	80.00%

**Fig 13:**
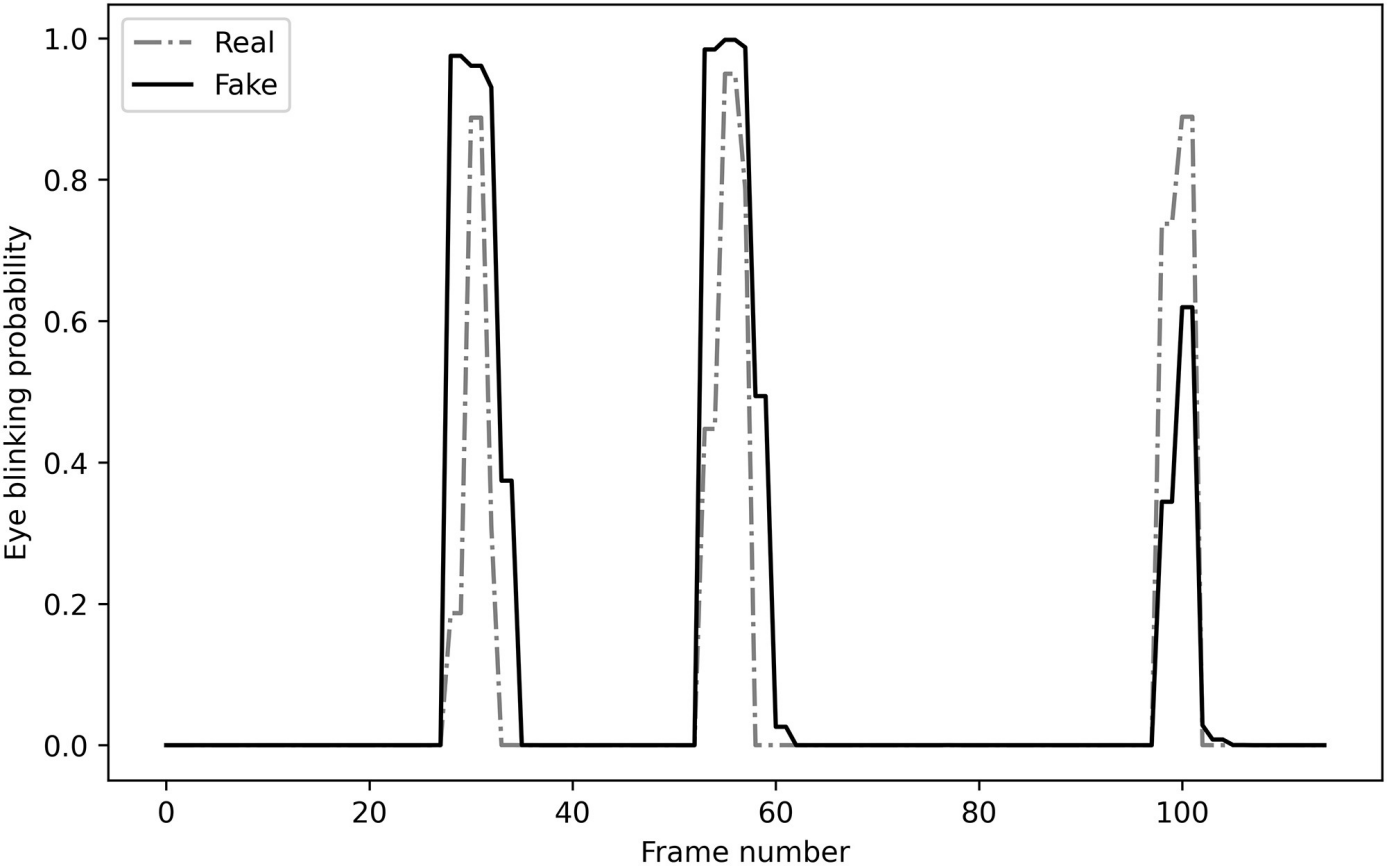
Comparison between real and fake eye blinking sequence for false prediction output.

In terms of overall performance, the model probability of failing to forecast the test video remained significant. Based on the model evaluation of false predictions, most cases occurred when the generated video generated an eye sequence that closely resembled the source. Fig 13 illustrates the relative similarity of the generated eye sequence with that of the original eye. Perceivably, the generated eye sequence between opening and closing states was more instantaneous and delayed than the original sequence despite a minute difference between them.

## Conclusion

The current study introduced a novel means of exposing fake videos created with deep neural networks, which depended on identifying eye blinking in videos: a physiological signal not typically included in fake videos. This approach, which was tested on datasets containing sequences that include eye-blinking, demonstrated optimal outcomes in detecting the fake videos created with the DeepFake-based programme. The method could distinguish between real and fake image sequences with up to 90.8% accuracy with Model 1 and a batch size of 20 at 105 epochs. DeepFakeDetection-generated media could be identified with up to 95.57% accuracy (the highest percentage) in the FaceForensics++ dataset, followed by DeepFakes, FaceSwap, and Face2Face at 94.65%, 91.54%, and 90.37%, respectively. Neural Textures-generated media denoted the lowest model accuracy performance at 86.76%.

The researchers intend to take this study in several distinctive directions for improved performance. First, alternative deep neural network architectures require further examination to determine the presence of more effective training methods to identify eye-blinking patterns. Second, more complex and complete rhythms of blinking, such as physiologically-impossible and excessive blinking that may indicate tampering could be included as the blinking state is the only input in the current method. Lastly, eye-blinking is merely a basic cue to detect fake face images as forgers would produce convincing blinking effects with post-processing and advanced models trained using blinking image sequence once the detection techniques gains popularity. Thus, it proves necessary to consider alternative physiological signals that could distinguish a real image sequence from those generated by AI synthesis methods.

The study is concluded as follows:

1. Using a novel dataset potentially accelerated the training process by lowering the calculation requirements and reducing the memory needed by the GPU in the training process.
2. By providing the eye state probability as the FCN model input, the trained model could provide optimal results with up to 90.80% accuracy.
3. Early stopping can provide good models faster and automatically but prevent the establishment of a better model, which could be derived by training over more epochs.
4. Based on the most complex model (Model 3), large batch size did not provide a significant improvement. Nevertheless, controlling the batch size proved pivotal for Models 1 and 2 with fewer hidden layers to ensure that they were not overfitting to the training and validation datasets.

## Future works

Significant advancements have been identified in multimedia forensics over the last 16 years. The establishment of new detection methods is a continuous process given the perpetuity of various unresolved issues and challenges. In this vein, deep learning catalysed the development of both media manipulation techniques and forensic technologies. An FCN proved suitable for classification with no specific assumptions on the inputs. Notwithstanding, an input with more dimensions or features would lead to an increase in the number of weights slow training time, and high GPU memory usage. Alternative classification techniques, such as CNNs could accelerate the process and minimize the possibility of overfitting during training. Overall, this study generated empirical results with time domain input features. By pre-processing the datasets to produce frequency domain features, additional temporal information could be provided to the training process for enhanced performance.
